# Differential Associations of Apolipoprotein E ε4 Genotype With Attentional Abilities Across the Life Span of Individuals With Down Syndrome

**DOI:** 10.1001/jamanetworkopen.2020.18221

**Published:** 2020-09-28

**Authors:** Hana D’Souza, Luke Mason, Kin Y. Mok, Carla M. Startin, Sarah Hamburg, Rosalyn Hithersay, R. Asaad Baksh, John Hardy, Andre Strydom, Michael S. C. Thomas

**Affiliations:** 1 Department of Psychology and Newnham College, University of Cambridge, Cambridge, United Kingdom; 2 Centre for Brain and Cognitive Development, Birkbeck, University of London, London, United Kingdom; 3 The London Down Syndrome (LonDownS) Consortium, London, United Kingdom; 4 Queen Square Institute of Neurology, Department of Neurodegenerative Disease, University College London, London, United Kingdom; 5 Division of Life Science, Hong Kong University of Science and Technology, Hong Kong SAR, People’s Republic of China; 6 Institute of Psychiatry, Psychology, and Neuroscience, Department of Forensic and Neurodevelopmental Sciences, King’s College London, London, United Kingdom; 7 Department of Psychology, University of York, York, United Kingdom; 8 UK Dementia Research Institute at University College London, University College London Institute of Neurology, Department of Neurodegenerative Disease, University College London, London, United Kingdom; 9 Reta Lila Weston Institute, Queen Square Institute of Neurology, University College London, London, United Kingdom; 10 National Institute for Health Research University College London Hospitals Biomedical Research Centre, London, United Kingdom; 11 Institute for Advanced Study, Hong Kong University of Science and Technology, Hong Kong SAR, People’s Republic of China; 12 South London and the Maudsley National Health Service Foundation Trust, London, United Kingdom

## Abstract

**Importance:**

Risk of Alzheimer disease (AD) is particularly high for individuals with Down syndrome (DS). The ε4 allele of the apolipoprotein E gene (*APOE *ε4) is associated with an additional risk for AD. In typical development, there is evidence that the *APOE *ε4 genotype is associated with an early cognitive advantage. Here we investigate associations of *APOE *ε4 with attention across the life span of individuals with DS.

**Objective:**

To investigate associations between *APOE *ε4 and attentional abilities in young children and in adults with DS.

**Design, Settings, and Participants:**

In this cross-sectional study, data were collected from 80 young children with DS (8-62 months of age) and 240 adults with DS (16-71 years of age) during the period from 2013 to 2018 at a research center to examine the association between *APOE* status (ε4 carrier vs ε4 noncarrier) and attentional abilities.

**Exposure:**

*APOE* status (ε4 carrier vs ε4 noncarrier).

**Main Outcomes and Measures:**

For the children, attentional ability was assessed using an eye-tracking paradigm, the gap-overlap task; the size of the gap effect was the primary outcome. For the adults, attentional ability was assessed using the CANTAB simple reaction time task; the standard deviation of response time latencies was the primary outcome. Cross-sectional developmental trajectories were constructed linking attentional ability with age in ε4 carriers and ε4 noncarriers for children and adults separately.

**Results:**

The child sample comprised 23 ε4 carriers and 57 ε4 noncarriers. The adult sample comprised 61 ε4 carriers and 179 ε4 noncarriers. For the children, a significant difference between trajectory intercepts (η_p_^2^ = 0.14) indicated that ε4 carriers (*B* = 100.24 [95% CI, 18.52-181.96]) exhibited an attentional advantage over ε4 noncarriers (*B* = 314.78 [95% CI, 252.17-377.39]). There was an interaction between *APOE* status and age (η_p_^2^ = 0.10); while the gap effect decreased with age for ε4 noncarriers (*B* = −4.58 [95% CI, −6.67 to −2.48]), reflecting the development of the attention system, there was no change across age in ε4 carriers (*B* = 0.77 [95% CI, −1.57 to 3.12]). For the adults, there was no main effect of ε4 carrier status, but there was an interaction between *APOE* status and age (*B* = 0.02 [95% CI, 0.004-0.07]), so that ε4 carriers had poorer attentional ability than ε4 noncarriers at older ages.

**Conclusions and Relevance:**

*APOE *ε4 is associated with an attentional advantage early in development and a disadvantage later in life for individuals with DS, similar to the pattern reported in typical development. Understanding the differential role of *APOE* across the life span is an important step toward future interventions.

## Key Points

**Question:**

What are the associations between the Alzheimer disease risk allele apolipoprotein E (*APOE*) ε4 and attention across the life span of individuals with Down syndrome?

**Findings:**

In a cross-sectional study including 80 young children and 240 adults with Down syndrome, an advantage was observed in attention for ε4 carriers relative to ε4 noncarriers among young children. Among young adults, no attentional advantage was observed in ε4 carriers, and possession of an ε4 allele was associated with a disadvantage among older adults.

**Meaning:**

Although *APOE *ε4 is a risk allele for Alzheimer disease later in life, it may be associated with an attentional advantage in the early development of individuals with Down syndrome.

## Teaser

This cross-sectional study investigates the associations between apolipoprotein E (*APOE*) ε4 genotype and attentional abilities across the life span of individuals with Down syndrome.

## Introduction

Individuals with Down syndrome (DS) show a much higher rate of Alzheimer disease (AD) than the general population, likely owing to the extra copy of chromosome 21.^1^ Yet, a large amount of variability in the clinical presentation and age at onset of AD exists among individuals with DS.^2^ Some of this variability may be explained by variation in the apolipoprotein E (*APOE*) gene on chromosome 19, with the ε4 allele of *APOE* associated with an increased risk for AD in both the general population and in individuals with DS.^2,3,4^

Even though AD emerges over the last few decades of life, changes associated with the different alleles of the *APOE* gene may already be detected in early development.^5,6,7^ Some studies of typically developing individuals suggest that the same ε4 allele that is associated later in life with AD risk may provide an advantage over other variants of the gene in early development (representing an example of genetic antagonistic pleiotropy).^7,8^ However, it is unclear what role *APOE* plays across the life span of an individual with DS, given that DS is associated with both intellectual disability in early life and an ultra-high risk for AD in later life.^1^

The *APOE* gene plays a central role in the metabolism of lipids,^8^ the principal components of myelin, and myelination is a crucial process in white matter development.^5,9^ The early development of white matter pathways has been associated with faster reaction times in an attentional eye-tracking task, the gap-overlap task.^10^ The ε4 allele has been associated with higher myelination levels during infancy but slower myelination across early childhood compared with non-ε4 alleles.^5,9^ We therefore hypothesized that developmental changes in attentional abilities (as a core cognitive function that can be measured across the life span) will reflect developmental changes in myelination, as well as cognitive decline associated with neurodegeneration due to AD in later life.

## Methods

### Participants

This study was embedded in the London Down Syndrome (LonDownS) Consortium, a large project aiming to understand the life-span development of individuals with DS. Of 115 younger children and 452 adults with DS, recruited via existing participant databases and support groups,^11^ genetic and attentional data were available for 81 children and 243 adults (for more details, see eMethods 1 in the Supplement). One child and 3 adults carried genotype *APOE* ε2/ε4, which has an unclear association with AD risk, and were therefore excluded from further analyses. The final sample consisted of 80 children and 240 adults with DS (Table).

**Table.** Characteristics of Participants

Characteristic	Children, No. (%)		Adults, No. (%)		Comparison, ε4 carriers vs ε4 noncarriers		*P* value, ε4 carriers vs ε4 noncarriers	
	ε4 Carriers	ε4 Noncarriers	ε4 Carriers	ε4 Noncarriers	Children	Adults	Children	Adults
No.	23	57	61	179	NA	NA	NA	NA
Age, median (IQR)	36.0 mo (20.0-43.0 mo)	24.0 mo (16.0-32.5 mo)	37.0 y (24.5-50.0 y)	40.0 y (26.0-50.0 y)	*U* = 838.50	*U* = 4993.50	.05	.32
*APOE* genotype								
ε2/ε2	0	0	0	3 (1.7)	NA	NA	NA	NA
ε2/ε3	0	11 (19.3)	0	34 (19.0)				
ε3/ε3	0	46 (80.7)	0	142 (79.3)				
ε3/ε4	21 (91.3)	0	58 (95.1)	0				
ε4/ε4	2 (8.7)	0	3 (4.9)	0				
Sex								
Female	11 (47.8)	21 (36.8)	30 (49.2)	84 (46.9)	χ^2^_1_ = 0.82	χ^2^_1_ = 0.09	.45	.76
Male	12 (52.2)	36 (63.2)	31 (50.8)	95 (53.1)				
Ethnicity^a^								
White	21 (91.3)	45 (78.9)	55 (90.2)	160 (89.4)	Fisher exact test = 2.54	Fisher exact test = 2.99	.70	.54
Asian	0	5 (8.8)	0	5 (2.8)				
Black	1 (4.3)	3 (5.3)	3 (4.9)	6 (3.4)				
Mixed	1 (4.3)	3 (5.3)	3 (4.9)	5 (2.8)				
Other	0	1 (1.8)	0	3 (1.7)				
Psychotropic medication^b^	0	0	15 (24.6)	31 (17.5)^c^	NA	χ^2^_1_ = 1.46	NA	.23

Abbreviations: *APOE*, apolipoprotein E; IQR, interquartile range; NA, not applicable.

^a^ The options were defined by the investigators based on the Office for National Statistics classifications and reported by parents or caregivers. Ethnicity was measured in this study to ascertain that the demographic characteristics of ε4 carriers and ε4 noncarriers were comparable.

^b^ Psychotropic medication as reported by parents or caregivers was compared across ε4 carriers and ε4 noncarriers because it may be associated with attentional performance.^23^

^c^ Report on psychotropic medication missing from 2 ε4 noncarriers in the adult sample.

Ethical approval was obtained for all adults and children from the North West Wales National Health Service Research Ethics Committee and for children from the Birkbeck Psychological Sciences Ethics Committee. Written informed consent was obtained from the parents of all of the children, from adults when they had the capacity to consent, and via an appointed consultee when the adults did not have the capacity to consent, in accordance with the UK Mental Capacity Act 2005. Participants were given a small gift in return for their participation. The Strengthening the Reporting of Observational Studies in Epidemiology (STROBE) reporting guidelines for cross-sectional studies were followed.

### Procedure and Materials

*APOE* genotype was determined using a Thermo Fisher Scientific Taqman assay for single-nucleotide variations rs7412 and rs429358 from saliva or blood samples.

The gap-overlap task was used to measure attentional ability in children, through assessing the efficiency of visual orienting.^12,13^ In this gaze-contingent eye-tracking task (eMethods 2 in the Supplement), the child was presented with a central stimulus (CS) followed by a peripheral stimulus (PS). Attentional abilities were probed in 3 conditions which manipulated the timing of these stimuli. In the baseline condition, CS offset occurred at the same time as PS onset. In the gap condition, CS offset preceded PS onset by 200 milliseconds. In the overlap condition, the CS remained on screen at PS onset and for the duration of PS presentation. Attentional abilities were assessed based on the difference between the saccadic reaction time (SRT) for the overlap and gap conditions, called the *gap effect*.^14^

The simple reaction time task from the Cambridge Neuropsychological Test Automated Battery (CANTAB) was used to measure attentional ability in adults.^15^ Participants were required to press a button as soon as a white square appeared on the computer screen (eMethods 3 in the Supplement). Response time in this task is dependent on both attentional and motor abilities, with the latter known to be affected with a high degree of variability in individuals with DS.^16^ To account for this variability, the standard deviation (SD) of the response time rather than the response time itself was used as the measure of attention. This allowed for an estimate of consistency in response time, and thus better reflected attentional ability than response time itself.^16^ Floor effects were managed in keeping with previous studies.^2^

### Statistical Analysis

General linear models were used to examine the association between *APOE *ε4 status and attentional abilities in children and adults with DS. The level of α was set to .05, and all tests were 2-tailed. *B* represents the unstandardized coefficients.

## Results

The child sample comprised 23 ε4 carriers (28.8%) and 57 ε4 noncarriers (71.3%), and the adult sample comprised 61 ε4 carriers (25.4%) and 179 ε4 noncarriers (74.6%) (Table). The prevalence of the *APOE *ε4 allele in the present study reflects the distribution in the general population.^4^

### Child Sample

**Figure 1.**
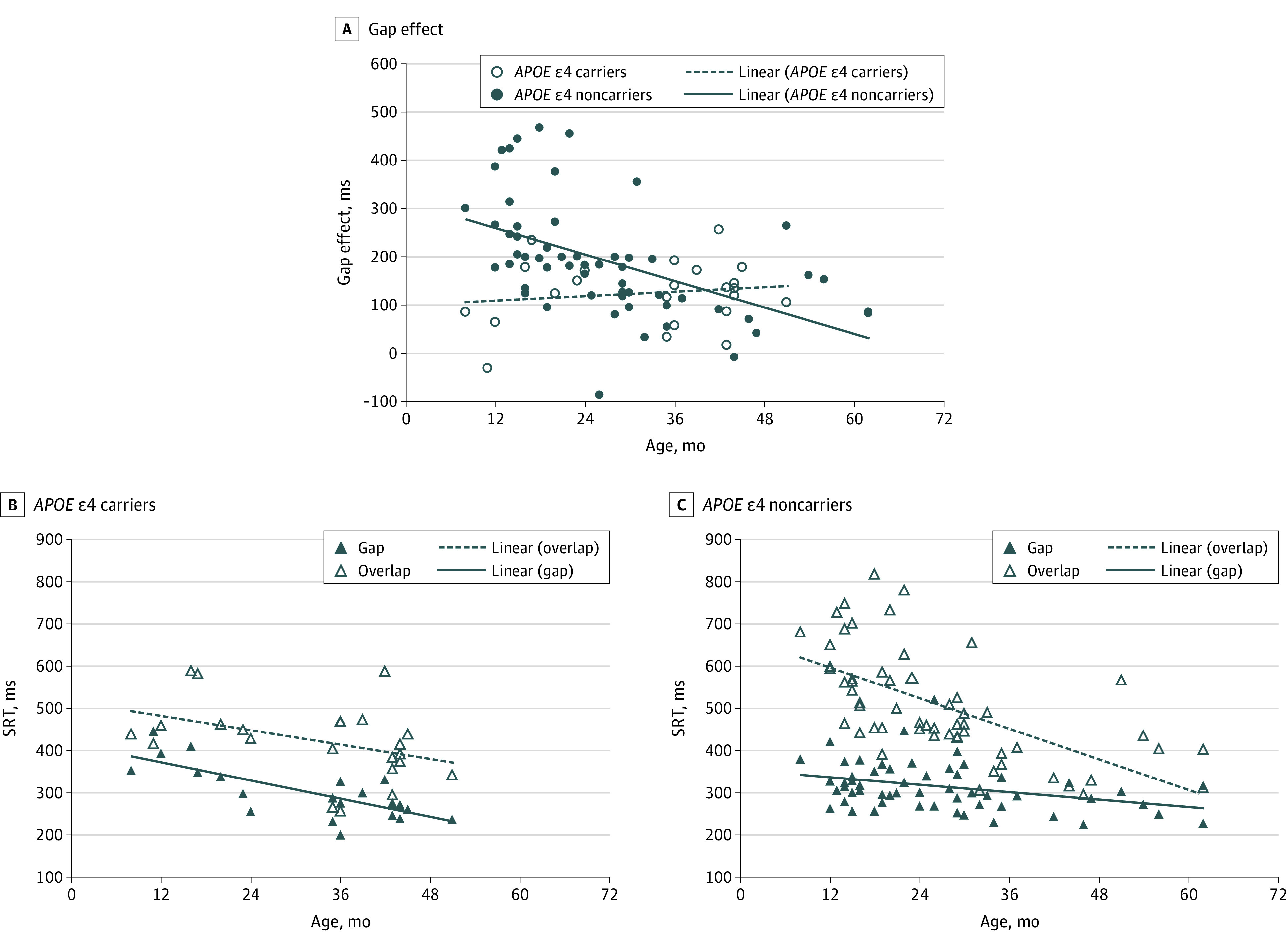
Child Sample: Associations Between Gap-Overlap Task and *APOE *ε4 Status A, Gap effect in ε4 carriers and ε4 noncarriers computed as gap saccadic reaction time (SRT) subtracted from overlap SRT. B, Gap SRT and overlap SRT in ε4 carriers. C, Gap SRT and overlap SRT in ε4 noncarriers.

The trajectories of the gap effect across age among children with DS who were ε4 carriers and ε4 noncarriers (Figure 1A) were compared using a general linear model estimating the gap effect from age with *APOE* group as a between-participants factor. As indicated by a significant difference between trajectory intercepts (*F*_1,76_ = 12.22; *P* < .001; η_p_^2^ = 0.14), ε4 carriers (*B* = 100.24 [95% CI, 18.52-181.96]; *P* = .02) exhibited an attentional advantage over ε4 noncarriers (*B* = 314.78 [95% CI, 252.17-377.39]; *P* < .001). While the gap effect decreased with age among ε4 noncarriers (*B* = −4.58 [95% CI, −6.67 to −2.48]; *P* < .001), it did not decrease with age among ε4 carriers (*B* = 0.77 [95% CI, −1.57 to 3.12]; *P* = .50; interaction of *APOE* group × age: *F*_1,76_ = 8.55; *P* = .005; η_p_^2^ = 0.10). Decompositions of trajectories for the gap condition (oculomotor efficiency) and overlap condition (attentional disengagement + oculomotor efficiency)^10^ are shown in Figure 1B and C. While the SRTs of the ε4 noncarriers decreased more rapidly in the overlap condition (*B* = −6.05 [95% CI, −8.10 to −3.99]; *P* < .001) than in the gap condition (*B* = −1.47 [95% CI, −2.55 to −0.39]; *P* = .009) (*F*_1,55_ = 19.10; *P* < .001; η_p_^2^ = 0.26), this was not the case for ε4 carriers (gap condition: *B* = −3.60 [95% CI, −4.99 to −2.22]; *P* < .001; overlap condition: *B* = −2.83 [95% CI, −5.68 to 0.01]; *P* = .05) (*F*_1,21_ = 0.47; *P* = .50; η_p_^2^ = 0.02). The slopes were significantly different between the groups in the gap condition (interaction of *APOE* group × age: *F*_1,76_ = 4.90; *P* = .03; η_p_^2^ = 0.06). In the overlap condition, the groups differed in intercepts (ε4 carriers: *B* = 516.51 [95% CI, 417.29-615.72]; *P* < .001; ε4 noncarriers: *B* = 669.42 [95% CI, 608.12-730.72]; *P* < .001) (*F*_1,76_ = 6.01; *P* = .02; η_p_^2^ = 0.07). Taken together, these results indicate an early attentional advantage but slower development in ε4 carriers compared with ε4 noncarriers.

### Adult Sample

**Figure 2.**
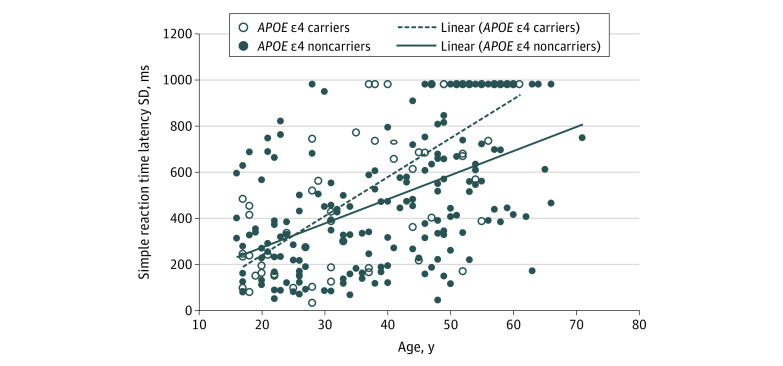
Adult Sample: Associations Between Cambridge Neuropsychological Test Automated Battery Simple Reaction Time Task and *APOE *ε4 Status Simple reaction time latency standard deviation (SD) in ε4 carriers and ε4 noncarriers for adult participants.

For the larger adult sample, a general linear model was used to estimate simple reaction time latency SD from age, *APOE* group, sex, and an *APOE* group × age interaction term (Figure 2). The regression model was significant (*F*_4,235_ = 28.03; *P* < .001; *R^2^* = 0.32). Simple reaction time latency SD increased with age, but there were no main effects of *APOE* group or sex (age: *B* = 0.06 [95% CI, 0.04-0.10]; *P* < .001; female sex: *B* = 0.66 [95% CI, −2.17 to 3.76]; *P* = .74; ε4 carriers: *B* = −20.04 [95% CI, −82.64 to 8.44]; *P* = .15). However, the *APOE* group × age interaction was significant (*B* = 0.02 [95% CI, 0.004-0.07]; *P* = .03), indicating a faster reduction of attentional abilities in ε4 carriers with increasing age; performance was poorer in the ε4 carrier group from midlife (Figure 2). A model that included level of intellectual disability explained more of the variance in adult attentional abilities but did not alter the pattern of effects of age and its interaction with *APOE* group (eResults in the Supplement).

## Discussion

To our knowledge, this is the first study showing that the ε4 variant of the *APOE* gene is associated with an advantage in early development in individuals with DS, a neutral effect in early adulthood, and a deleterious effect in late adulthood (Figure 1 and Figure 2). The early advantage is consistent with a report on typically developing 2-year-olds when ε4 carriers performed better on a standardized developmental test (the Bayley Scales of Infant Development, Second Edition) than ε4 noncarriers,^7^ whereas cognitive studies of older children and young adults typically do not show differences between ε4 carriers and ε4 noncarriers.^17^ The poorer attentional abilities observed in late adulthood in ε4 carriers compared with ε4 noncarriers are likely associated with the increased risk of AD for ε4 carriers.^3^

In our child sample, we observed an early attentional advantage but slower development in ε4 carriers compared with ε4 noncarriers. These results are consistent with findings for typical development that ε4 carriers between 2 and 25 months of age show greater myelination early in development than ε4 noncarriers but subsequent slower development.^5,9^ However, the exact nature of the neural correlates associated with attentional trajectories in early development, and how these are associated with *APOE* status, remains to be fully examined. In adults, the visuospatial attentional orienting system is underpinned by a functional network that includes left and right parietal regions, and is linked to a larger neural network including frontal eye fields and subcortical areas including the superior colliculus.^18^ Frontal and parietal regions have been found to have a higher myelin water fraction in 2- to 6-month-old ε4 carriers,^5^ potentially contributing to the early attentional advantage observed in the present study. Furthermore, in infants and adults, the splenium of the corpus callosum has been identified as a large white matter region that can be used as a marker of individual differences in the orienting network and associated attentional performance (particularly on the overlap trials of the gap-overlap task).^10,19^ The association between age and myelin water fraction of the splenium is attenuated in ε4 carriers early in development.^5^ This is in line with our findings of an attenuated association between age and the gap effect (likely due to performance on overlap trials that emphasize attentional disengagement) in ε4 carriers. In later life, even though the splenium of the corpus callosum may not be the primary area associated with AD, the rate of atrophy of this region has been found to be associated with the progression of AD severity, possibly owing to a loss of callosally projecting cortical neurons.^20^

The increased risk of AD for ε4 carriers is likely associated with the poorer attentional abilities that we observed in late adulthood in ε4 carriers compared with ε4 noncarriers on the CANTAB simple reaction time task. Using the same adult sample reported here, this task has been found to be one of the most sensitive tasks associated with cognitive decline in adults with DS.^2^ In the present study, we detected diverging trajectories on this task based on *APOE* status, consistent with the onset of AD brain pathology in individuals with DS in their 30s.^1^ Thus, our results are in line with findings that the *APOE *ε4 genotype is associated with an earlier and faster progression of AD.^2,3^

Taken together, our results could be viewed as the differential effects of *APOE *ε4 across the life span (an antagonistic pleiotropic effect). However, it remains unclear how *APOE* involvement in early development is mechanistically connected to AD. Several nonmutually exclusive hypotheses have been advanced for the mechanisms by which *APOE *ε4 increases risk of AD, based on the role of *APOE* in lipid metabolism. These include enhancement of amyloid-β production, modulation of tau phosphorylation, increased deposition of transactive response DNA-binding protein 43, reduction of lipid metabolism, accentuated mitochondrial dysfunction, higher susceptibility to neuroinflammation, reduction of vascular integrity, disruption to insulin and vascular endothelial growth factor signaling, and disruptions to synaptic plasticity and repair.^21^ With respect to plasticity, in particular, it has been suggested that possession of the ε4 allele is associated with higher levels of synaptic macromolecular turnover, which may facilitate early development but also may stress basic cellular neuroplasticity mechanisms.^8^ This would explain the improved early performance at the expense of a decreased performance during aging.^22^

### Limitations

Even though the current sample size enabled us to investigate differences between ε4 carriers and ε4 noncarriers, it was not large enough to probe associations of attention and individual *APOE* genotypes. These include life-span investigations associated with the dose effect of ε4 (ε3/ε4 vs ε4/ε4); the role of the AD-protective ε2 variant early in development; and the performance of ε2/ε4 carriers, because the combined effect of possessing both the AD-protective ε2 variant and the AD-risk ε4 variant is unclear.

Furthermore, we used 2 different age-appropriate measures to assess attention. Associations of *APOE* with different subtypes of attention, as well as different aspects of cognition more broadly, remain to be investigated to understand whether ε4 is associated with a general early cognitive advantage.

## Conclusions

In this study, *APOE *ε4 was associated with an attentional advantage early in development and a disadvantage later in life in individuals with DS, similar to the pattern reported in typical development. Understanding the differential role of *APOE* over the life span is an important step toward targeting interventions based on a better understanding of the risk and protective factors for life-span development in individuals with DS and in other individuals at risk of AD.

## Notes

Supplement. **eMethods 1.** Participants **eMethods 2.** The Gap-Overlap Task **eMethods 3.** CANTAB Simple Reaction Time Task **eResults.** Impact of Intellectual Disability on Simple Reaction Time Latency Standard Deviation **eReferences.**
