# Errors in Panels B and C in Figure 1

**DOI:** 10.1001/jamanetworkopen.2020.30697

**Published:** 2020-12-01

In the Original Investigation titled “Differential Associations of Apolipoprotein E ε4 Genotype With Attentional Abilities Across the Life Span of Individuals With Down Syndrome,”^1^ published online September 28, 2020, the dashed and solid lines and their respective labels in panels B and C of Figure 1 should be switched. This article has been corrected.
