# Supplementary material for: Differential Associations of Apolipoprotein E ε4 Genotype With Attentional Abilities Across the Life Span of Individuals With Down Syndrome
Source: JAMA Netw Open. 2020 Sep 28;3(9):e2018221. doi: 10.1001/jamanetworkopen.2020.18221 (PMC7522696; doi:10.1001/jamanetworkopen.2020.18221)
Supplement: Supplement. — eMethods 1. Participants eMethods 2. The Gap-Overlap Task eMethods 3. CANTAB Simple Reaction Time Task eResults. Impact of Intellectual Disability on Simple Reaction Time Latency Standard Deviation eReferences. [file jamanetwopen-e2018221-s001.pdf]

Supplementary Online Content

D'Souza H, Mason L, Mok KY, et al; London Down Syndrome (LonDownS) Consortium. Differential associations of apolipoprotein E ε4 genotype with attentional abilities across the life span of individuals with Down syndrome. *JAMA Netw Open*. 2020;3(9):e2018221. doi:10.1001/jamanetworkopen.2020.18221

eMethods 1. Participants
eMethods 2. The Gap-Overlap Task
eMethods 3. CANTAB Simple Reaction Time Task
eResults. Impact of Intellectual Disability on Simple Reaction Time Latency Standard Deviation
eReferences.

This supplementary material has been provided by the authors to give readers additional information about their work.

© 2020 D'Souza H et al. *JAMA Network Open.*

eMethods 1. Participants

Out of 115 young children with Down syndrome (DS) recruited via existing participant databases and support groups (see[1]), 104 attended the lab session and 98 performed an attentional eye-tracking task. Insufficient data quality (see eMethods 2 in the Supplement) led to the exclusion of 14 children, and genetic data were not available for three children. Of the remaining 81 children, one child carried genotype *APOE* ε2/ε4, which has an unclear association with AD risk, and was excluded from further analyses. The final sample therefore consisted of 80 children aged between 8 and 62 months. Twenty-three children (28.8%) were ε4 carriers and 57 (71.3%) were ε4 noncarriers, which reflects the distribution in the typically developing population.[2] The *APOE* groups did not differ in key participant characteristics (Table).

The adult group consisted of 452 participants with DS, recruited through similar channels as the children group (see[1,3]). Genetic and attentional data of interest were available for 243 individuals. Three participants carried *APOE* ε2/ε4 and were excluded from any further analysis. The final sample of 240 participants consisted of adults aged between 16 and 71 years, with 61 (25.4%) ε4 carriers and 179 (74.6%) ε4 noncarriers. This is similar to the child group and the typically developing population.[2] Again, the *APOE* groups did not differ in key participant characteristics (Table).

eMethods 2. The Gap-Overlap Task

This gaze-contingent task was a part of a larger battery of tasks and was adopted from the British Autism Study of Infant Siblings (BASIS). It has previously been successfully used with young children with DS.[4] The task was administered using a Tobii TX300 eye-tracking system (Tobii AB, Stockholm, Sweden). The participants were seated in front of a 23-inch liquid-crystal display (LCD) monitor at a distance of around 65cm. A webcam was used to monitor behaviour, and sessions were video recorded for analytic reference. Stimulus presentation and data collection were performed using the Task Engine (https://sites.google.com/site/taskenginedoc/) framework for Matlab (MathWorks, Inc., Natick, Massachusetts, United States).

An automatic five-point calibration was run wherein coloured spirals expanded and contracted in each of the four corners and in the centre of the screen. Following this brief five-point calibration, participants completed the gap-overlap task (adapted from[5,6]). In this task, participants were presented with a gaze-contingent central stimulus (CS; i.e., a dynamic colourful clock) and, subsequently, a peripheral stimulus (PS; i.e., a white cloud) which appeared randomly either to the right or left of the CS at an eccentricity of 19°. The CS initially loomed on screen until the participant fixated on it, at which point it span for a random 600-700ms in order to ensure attentional engagement before the onset of the PS. When the participant fixated upon the PS, or 1200ms after PS onset (whichever was sooner), the reward stimulus was presented. This took the form of various cartoon characters at the location of the PS which span or shrank whilst emitting various child-friendly sounds. Saccadic reaction times (SRTs) were calculated as the latency in ms to disengage from the CS and saccade to the PS.

Attentional abilities were probed in three conditions which manipulated the timing of the offset of the CS. In the baseline condition, CS offset occurred at the same time as PS onset. In the gap condition, CS offset preceded PS onset by 200ms. In the overlap condition, the CS remained on screen at PS onset and for the duration of PS presentation. Trials were presented in blocks of 12, until 12 valid trials per condition were acquired or a maximum of 60 trials were presented. Trials were considered valid if the following conditions were met: (1) gaze fell on the CS; (2) no gaps of missing data longer than 200ms were present during the CS period (before PS onset); (3) there was at least one sample of gaze at the CS area of interest within 50ms either side of PS onset; (4) no gaps of missing data longer than 100ms were present during the PS period (between PS onset and reward onset); (5) SRT was longer than 150ms and shorter than 1200ms; (6) gaze did not go in the opposite direction to the side of the PS; (7) gaze did not enter the PS area of interest (AOI) after engagement with the CS but before PS onset. Trials that failed any of the above criteria were invalidated and removed from further analysis. Mean SRTs were calculated for each condition separately, using only valid trials. Participants with fewer than six valid trials per condition were removed entirely from the analysis. For the purpose of the current research question, two conditions of this task were of interest for the analysis: gap and overlap. The *gap effect* was computed by subtracting gap SRT from overlap SRT.[7]

eMethods 3. CANTAB Simple Reaction Time Task

For this task there was a variable stimulus onset asynchrony (SOA) of between 750ms and 1500ms and a pre-empt delay of 100ms in which any response was regarded as 'too early'. Following the pre-empt delay, participants had 3000ms to respond to each trial. Participants completed an initial practice block of 24 trials, followed by two test blocks of 50 trials each. The standard deviation (SD) of response times for these two test blocks was used as the outcome for this task.

eResults. Impact of Intellectual Disability on Simple Reaction Time Latency Standard Deviation

To examine the added influence of level of intellectual disability on this relationship, we added this variable to the regression model with an *APOE* group*level of intellectual disability interaction to further examine the results found in the adult group. This regression model was significant, $F(8,231)=29.63$, $p<.001$, $R^2=.51$, with age ($B=0.06$ (95% CI [0.04, 0.09]), $p<.001$), female gender ($B=2.64$ (95% CI [0.48, 8.38]), $p=.030$), moderate level of learning disability ($B=12.45$ (95% CI [3.83, 26.58]), $p<.001$), severe level of learning disability ($B=95.20$ (95% CI [53.94, 148.36]), $p<.001$) and the *APOE* group*age interaction ($B=0.02$ (95% CI [0.003, 0.05]), $p=.046$) all showing significant associations with simple reaction time latency standard deviation. The *APOE* group*moderate intellectual disability interaction ($B=9.09$ (95% CI [-3.12, 0.05]), $p=.122$), *APOE* group*severe intellectual disability interaction ($B=-7.50$ (95% CI [-42.9, 21.98]), $p=.680$), and *APOE* group ($B=-19.41$ (95% CI [-76.51, 6.71]), $p=.123$) were not associated with performance. This model with intellectual disability was a better fit of the data ($p<.001$). These results show that *APOE* ε4 status is associated with attentional abilities as people age, even when adjusting for the impact of intellectual disability.

eReferences

1. Startin CM, D'Souza H, Ball G, et al. Health comorbidities and cognitive abilities across the lifespan in Down syndrome. *J Neurodev Disord*. 2020;12(1):4. doi:10.1186/s11689-019-9306-9
2. van der Lee SJ, Wolters FJ, Ikram MK, et al. The effect of APOE and other common genetic variants on the onset of Alzheimer's disease and dementia: a community-based cohort study. *Lancet Neurol*. 2018;17(5):434-444. doi:10.1016/S1474-4422(18)30053-X
3. Startin CM, Hamburg S, Hithersay R, et al. Cognitive markers of preclinical and prodromal Alzheimer's disease in Down syndrome. *Alzheimer's Dement*. 2019;15(2):245-257. doi:10.1016/j.jalz.2018.08.009
4. D'Souza D, D'Souza H, Jones EJH, Karmiloff-Smith A. Attentional abilities constrain language development: A cross-syndrome infant/toddler study. *Dev Sci*. 2020:e12961. doi:10.1111/desc.12961
5. Elsabbagh M, Fernandes J, Webb SJ, et al. Disengagement of Visual Attention in Infancy is Associated with Emerging Autism in Toddlerhood. *Biol Psychiatry*. 2013;74(3):189-194. doi:10.1016/j.biopsych.2012.11.030
6. Landry R, Bryson SE. Impaired disengagement of attention in young children with autism. *J Child Psychol Psychiatry*. 2004;45(6):1115-1122. doi:10.1111/j.1469-7610.2004.00304.x
7. Cousijn J, Hessels RS, Van Der Stigchel S, Kemner C. Evaluation of the Psychometric Properties of the Gap-Overlap Task in 10-Month-Old Infants. *Infancy*. 2017;22(4):571-579. doi:10.1111/infa.12185

© 2020 D'Souza H et al. *JAMA Network Open.*
